# Clinical Remarks upon Surgical Cases in the Buffalo General Hospital—Varicose Veins—Exsection of the Head of the Humerus

**Published:** 1865-11

**Authors:** J. F. Miner


**ART. III.—*Clinical Remarks upon Surgical Cases in the Buffalo General Hospital—Varicose Veins—Exsection of the head of the Humerus.*** By J. F. Miner, M. D.

*Varicose Veins.*—The first patient which I introduce to you is a relic of the Andersonville prison—that charnel-house—that Southern "Golgotha," which will forever stand as a blot of infamy and shame, only rendered deeper and more damnable by every attempt to conceal its guilt. His earnest and impartial recital of its horrors, united with half-suppressed emotions at the recollection of past sufferings and scenes of suffering, are sufficient to convince the most charitable, that at Andersonville, barbarity and inhumanity, reached a degree lower, than ever before known, among either men or devils. He was robbed and kept nine months, naked, without shelter, almost without food, often in the mud, suffering a hundred deaths himself, and seeing thousands of others die from want and disease. This history is so remarkable that I have thought it worthy a notice in connection with the operation we propose for the radical cure of varicose veins. It will serve to fasten in your recollections the fact that varicose veins are sometimes caused by hardships, exposures and fatigues; and it will also impress you with a just abhorrence and detestation of the inhuman brutes who could perpetrate such crimes.

I have for several years been in the habit of obliterating dilated veins, by injecting into them a solution of persulphate of iron; the manner of operating hardly requires a description. With a subcutaneous syringe, three drops of the solution, with about thirty of water, are injected into the main trunks of dilated or varicose veins; this may be done at different points. It produces an immediate coagulation of the blood, and thus the current is perfectly interrupted, the vessel commences to contract, and is soon only a mere cord, while the blood is obliged to circulate through the smaller and deeper vessels. It is the same in effect as ligating the vessel, an operation which is often resorted to with the same view. Obstruction is also sometimes obtained by placing over the dilated vessel a caustic issue; other modes of procedure have been practiced by surgeons and generally abandoned.

This plan of injecting solutions of iron into veins to coagulate the blood—of making internal obstruction of the circulation, and thus causing obliteration, is comparatively a new method of curing varicose veins. It has been repeatedly illustrated before the different classes, for the past few years, that this manner of procedure is safe and successful—safe, so far as any dangerous inflammation of veins is concerned, and perfectly successful in obstructing the current of blood and curing any varicose ulcers which may be present. It has been attempted by some excellent surgeons and condemned, because of the liability to induce extensive ulcers, the solution being thrown into the cellular tissue, around the vessel, or by danger of inflammation of the veins; the possibility also of not obstructing the circulation has been urged as an objection.

With no desire to induce others to adopt this plan, who think any other better, it is yet proper for me to say, that if the operation is made as shown to you—if the vessel is opened down upon with careful touches of the scalpel, until its blue walls are plainly exposed, the point of the syringe carefully introduced into the vessel, and no where else, and if the solution is reduced, and not used stronger than one drop of solution of persulphate to ten drops of water, not one of the objections urged against this operation can be sustained; in other words, the operator is in fault, while the plan itself is believed to be unsurpassed in excellence—practiced properly, it is invariably successful and satisfactory. I

have before described to some of you this operation, and perhaps spoken less positively than now of its merits; abundant experience has convinced me that it is an operation which can certainly be made with success and safety.*

*Exsection of the Head of the Humerus.*—Patrick K——, aged 55, healthy until the last two years, during which time he has suffered from pain, tumefaction and discharge of pus from the orifices you observe, which lead to the bone, but through which I have been unable to determine the exact nature on seat of disease; without exploratory incision it is impossible to decide the condition of the joint surfaces, or that this pus comes from inflammation and ulceration here, or is the result of caries or necrosis of the bone below the articulating surface. The patient has suffered great pain; has become pale and anæmic, and there is a presumption that the joint is diseased.

With the view of positively deciding if the shaft is the seat of disease, before opening the joint, an exploratory incision is made. By this means we have determined the lesion, we can now trace the disease to the articulating surfaces of the joint, and therefore proceed as proposed, to exsect the head of the humerus, with about three inches of the shaft, thus removing the entire disease of the bone.

Formerly such an operation would not be proposed, but instead, amputation at the shoulder-joint. If this operation then should prove successful in removing the disease, and still leaving the arm in any degree useful, it is certainly a great advance, in conservative surgery. To show what may sometimes be gained in this way, over amputation, I might remind you of a similar case which some will recollect, where the patient obtained a good arm and pursued his trade as a mechanic with acceptance. It is possible then, in favorable cases, to preserve a useful arm, by what has been called conservatism—by making exsection rather than amputation.

You have observed the pale, anæmic, unhealthy countenance of this patient, and you will perhaps ask if it has been induced by the discharge. Our patient has suffered from this diseese of the joint for two years, and from this ulceration of bone has escaped a

* This patient left the Hospital twenty days after the operation, perfectly relieved of both varicose veins and ulcers, which had resisted treatment for six months under the care of an experienced surgeon. This result is mentioned with the view to place the fact on record, hoping to contribute additional evidence in favor of this operation.

great amount of pus. The pain, loss of appetite, loss of sleep, and other causes have aided in producing the debility, but it is not certain that the pus secreted contributed much to the result, the other causes if present in equal degree, unaccompanied by pus formation, would have proved sufficient; but it is not easy to determine the influence of the different causes separately considered. One other question it would also be interesting to consider, viz: What was the cause of the disease? did it arise from general or constitutional bias, or was it induced by injury or other local causes? He knows of no local injury, and believes that it "came of itself," though we are not positive that he did not suffer injury possibly so slight and so long previous to manifest disease that it had escaped all recollection, yet had influence in inducing the disease; however this might have been, it is difficult to altogether exclude the belief in constitutional bias. If it was traumatic in this case, it does not follow that such disease is always induced by local or external causes; it is safer, to be slow in adopting as our own, any of the various theories which have been proposed.

